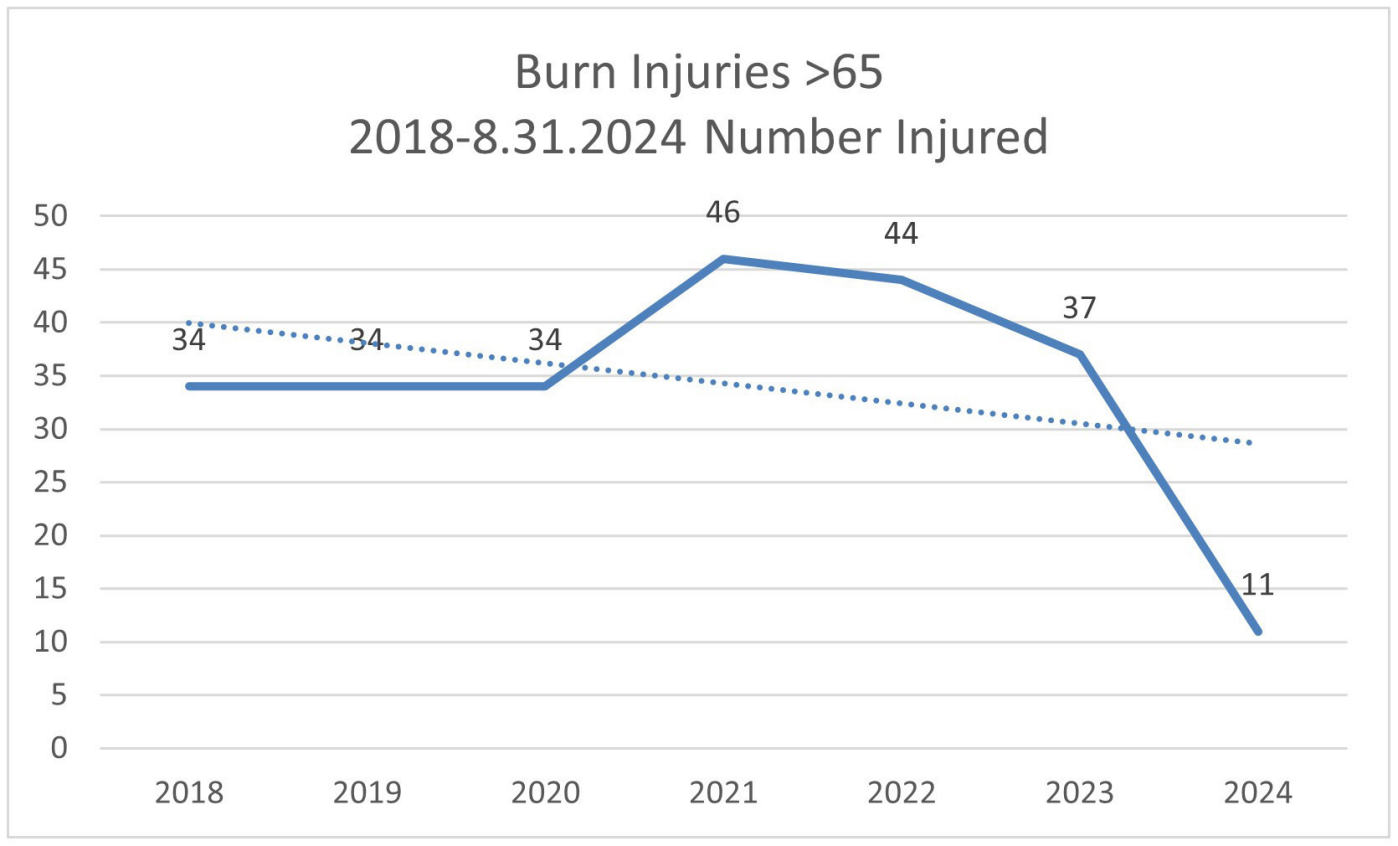# 119 Burn Prevention Outreach for Older Adults Using County Based Meal Delivery Programs

**DOI:** 10.1093/jbcr/iraf019.119

**Published:** 2025-04-01

**Authors:** Kara Judd, Tamara Roberts, Julia Guarrera

**Affiliations:** Clark Burn Center; Clark Burn Center; Clark Burn Center

## Abstract

**Introduction:**

Our burn center treats serious burn injuries in patients of all ages in a 38-county region of New York State.

We noted an alarming upward trend in injuries of persons 65 and older in 2021, which correlated with a lack of education being provided to this age group due to the COVID 19 pandemic. This age group was difficult to reach for community outreach during quarantine, and even after, as many in this age group were reluctant to gather in public places due to health concerns. The 65 and older population is often underserved, and has a higher morbidity and mortality rate related to burn injuries.

**Methods:**

Utilizing the existing home meal delivery service of the county Meals on Wheels programs, Clark Burn Center was able to provide 4,385 education-based burn prevention kits to elderly residents.

**Results:**

The program started in 2022, and we have seen a 67% decrease of burn injured adults ages 65 and older being treated in our burn center since 2018.

**Conclusions:**

By providing education to a hard to reach demographic, we have seen a downward trend in burn injuries in that demographic.

**Applicability of Research to Practice:**

Through our research on looking at our most vulnerable patients of burn injuries we discovered the elderly population comprised a large portion. The elderly often suffer worse from injury and the increase in preventable burn injuries following COVID was reason to create a program to try and decrease this number.

Wanted to include existing community resources that reach out to a large number of people and are well trusted in the community. Created this set of information packets with the elderly in mind with easy-to-read literature and easy to use items as well.

Through this program and tracking the results we have seen a noticeable decrease in injuries after implementation. This shows that prevention programs like this are effective in reducing injuries and further research could be done to expand our geographic scope as well as the long-term effects of the program.

These proactive measures help prevent burn injuries in the elderly population leading to a better quality of life and create safer home environments. In practice, partnership with community resources can be a beneficial way to reach large groups of people and provide tailored care to the groups enhancing the quality of care we provide.

**Funding for the Study:**

N/A